# Editorial: Artificial intelligence in digital pathology image analysis

**DOI:** 10.3389/fbinf.2023.1007986

**Published:** 2023-04-13

**Authors:** Yi Liu, Xiaoyan Liu, Hantao Zhang, Junlin Liu, Chaofan Shan, Yinglu Guo, Xun Gong, Min Tang

**Affiliations:** ^1^ School of Life Sciences, Jiangsu University, Zhenjiang, Jiangsu, China; ^2^ Institute of Animal Science, Jiangsu Academy of Agricultural Sciences, Nanjing, China; ^3^ Affiliated Hospital of Jiangsu University, Zhenjiang, China

**Keywords:** artificial intelligence, digital pathology, disease diagnosis, interpretable deep learning, multi-modality, precision medicine

In the 21st century, cancer is the top cause of death in hospitals and the key limitation of life expectancy in most countries (Luo et al.). The analysis of medical images including histopathological slides, radiological images such as magnetic resonance imaging (MRI) and CT, and ultrasound images, etc. is an essential tool in cancer research, disease diagnosis and treatment. Moreover, the availability of faster networks and cheaper storage solutions make these images easier to manage and share, leading to the emergence of digital pathology images, for example, whole slide imaging (WSI). However, extracting important information from these images for clinical use requires a big effort from pathologist and is also error-prone due to inexperience and fatigue. Recently, Artificial intelligence (AI) such as deep learning (DL) shows clear potential to mine image features from medical images, better quantitative model disease appearance and hence possibly improve prediction of disease aggressiveness and patient outcome. The application of AI not only reduces the burden on pathologists but also saves high costs and time, thus attracting great attention.

In this editorial, we presented an account of how AI has greatly facilitated digital pathology image analysis as well as other medical image analysis. This editorial is based on 11 research articles, 1 regular review and a methods article, shedding light on the power of AI to analyze medical images including but not limited to magnetic resonance imaging (MRI), CT images, and digital pathology images, primarily WSI.

Five research articles use machine learning to construct prediction models based on pathological and radiological images. Wang D. et al. developed an automated machine-learning framework for predicting IDH1 mutation status in glioma. In their framework, a random forest algorithm is applied to select relevant features in regions of interest (ROIs) extracted from high-resolution pathology slides and multi-sequence MRI scans. The model integrating histopathological and radiological information can predict glioma IDH genotype with greater accuracy and reliability (Wang D. et al.). Zhao F. et al. used random forest combined with hyperparameter tuning for feature selection and radiomics prediction modeling to distinguish invasive adenocarcinoma (IAC) and minimal invasive adenocarcinoma (MIA) presenting as ground-glass nodules (GGNs). The result of ROC curve showed that their model effectively distinguished IAC from MIA presenting as GGNs and represented a non-invasive, low-cost, rapid, and reproducible preoperative prediction method for clinical application (Zhao F. et al.). In Wang X. et al.’s study, Mann–Whitney *U* test and least absolute shrinkage and selection operator (LASSO) were applied for feature preselection and radiomic signature construction based on CT. SVM-linear models were trained by incorporating the radiomic signature with clinical characteristics. Importantly, they chose the optimal model to build a nomogram which could be useful to preoperatively predict histologic grade in pancreatic neuroendocrine tumors (Wang X. et al.). Hu et al. also utilized LASSO to select radiomics signatures. The Logistic algorithm and a combinatorial modeling approach were used to establish unimodal radiomics models and multimodal radiomics models respectively based on tumors and peritumors extracted from enhanced MRI images. The radiomics signatures of the dual regions for tumor and peritumor were fund to be of significance to predicty microvascular invasion risk grades in hepatocellular carcinoma preoperatively (Hu et al.). Furthermore, through multivariate logistic regression analysis and statistical analysis, Luo et al. found that shear wave elastography (SWE) examination, a newly emerging elastography technique which can display tissue stiffness in a quantified form to obtain the biological information of the primary lesion, can be used as a routine auxiliary method of conventional ultrasonic examination for axillary node metastasis and the elastic modulus values of SWE had no significant correlation with the molecular types of breast cancer (Luo et al.).

Several research articles focus on the application of deep-learning in image analysis for detection, feature extraction, and tissue classification. Based on whole slide imaging, Tao et al. used deep learning (DL) including AlexNet, VGG-16, Inception V3, DenseNet-121, ResNet-50, and MnasNet to classify bone tumors histopathologically in terms of aggressiveness. The results showed that DL can effectively classify bone tumors similar to senior pathologists, which is promising and would help expedite the future application of DL-assisted histopathological diagnosis for bone tumors (Tao et al.). The most of traditional DL models were also applied in Guo et al.’s study for automatically detecting circulating tumor cell (CTC) which is a critical biomarker for cancer diagnosis and prognosis based on immunofluorescence *in situ* hybridization (imFISH) images. Additionally, they used transfer learning to improve the prediction performance and save computing resources. Both DL and transfer learning detected CTCs with high sensitivity (Guo et al.). Xu Y. et al. evaluated DL model for predicting tumor invasiveness of ground-glass nodules (GGNs) through analyzing time series CT images (baseline CT and 3-month follow-up CT images). They also evaluated the effect of different ROIs on prediction. The DL model integrating full ROIs that contain both tumor and peritumor regions from serial CT images showed improved predictive performance, which could benefit the clinical management of GGNs (Xu Y. et al.). Moreover, Berberine was found to Suppress stemness and tumorigenicity of colorectal cancer stem-like cells by inhibiting m6A methylation in Zhao Z. et al.’s study by experiment (Zhao Z. et al.).

Notably, four articles proposed novel DL models to better analyze medical images based on existing algorithm. Luan et al. proposed a neural network (S-Net) which obtained more semantic information with the introduction of an attention mechanism and long jump connection, thus effectively improving the effect of liver tumors’ automated segmentation from CT images (Luan et al.). Xu F. et al. built a DL core-needle biopsy (DL-CNB) model on the attention-based multiple instance-learning frameworks to predict axillary lymph node metastasis in early breast cancer utilizing the DL features, which were extracted from the cancer areas of WSIs of breast CNB specimens annotated by two pathologists. And the interpretation of DL-CNB model showed that the top signatures most predictive of ALN metastasis were characterized by the nucleus features (Xu F. et al.). Based on the Residual Network (ResNet) model, Liu et al. proposed a deep learning method, DeepHE. On images of tissue, DeepHE can efficiently identify and analyze characteristics of tumor cells to predict the Tumor mutational burden (TMB) avoiding whole-exome sequencing which is a standard but costly and inefficient method to measure TMB (Liu et al.). Yang et al. proposed a novel deep learning framework to predict the benign and malignant thyroid nodules accurately. They first trained a ResNet18 model by an ultrasound image dataset. Gradient-weighted Class Activation Mapping (Grad-CAM) was then proposed to highlight sensitive regions, extracting the sensitive regions and analyzing their shape features. Shape features of the sensitive regions were helpful in diagnosis to a great extent (Yang et al.).

Finally, the review article summarized process and key steps of current pathological image processing including image preprocessing, image segmentation, feature extraction and model construction, to help researchers choose more suitable medical image processing methods and predict cancer-related biomarkers more accurately (Xie et al.).

In conclusion, the articles in this Research Topic show how AI is applied to analyze medical images including digital pathology images. For fully utilizing data from various pathological and radiological images to gain insights into disease, the applications of AI are becoming more relevant every day.

